# Supplementary figures and images for: KDM6B Variants May Contribute to the Pathophysiology of Human Cerebral Folate Deficiency
Source: Biology (Basel). 2022 Dec 31;12(1):74. doi: 10.3390/biology12010074 (PMC9855468; doi:10.3390/biology12010074)

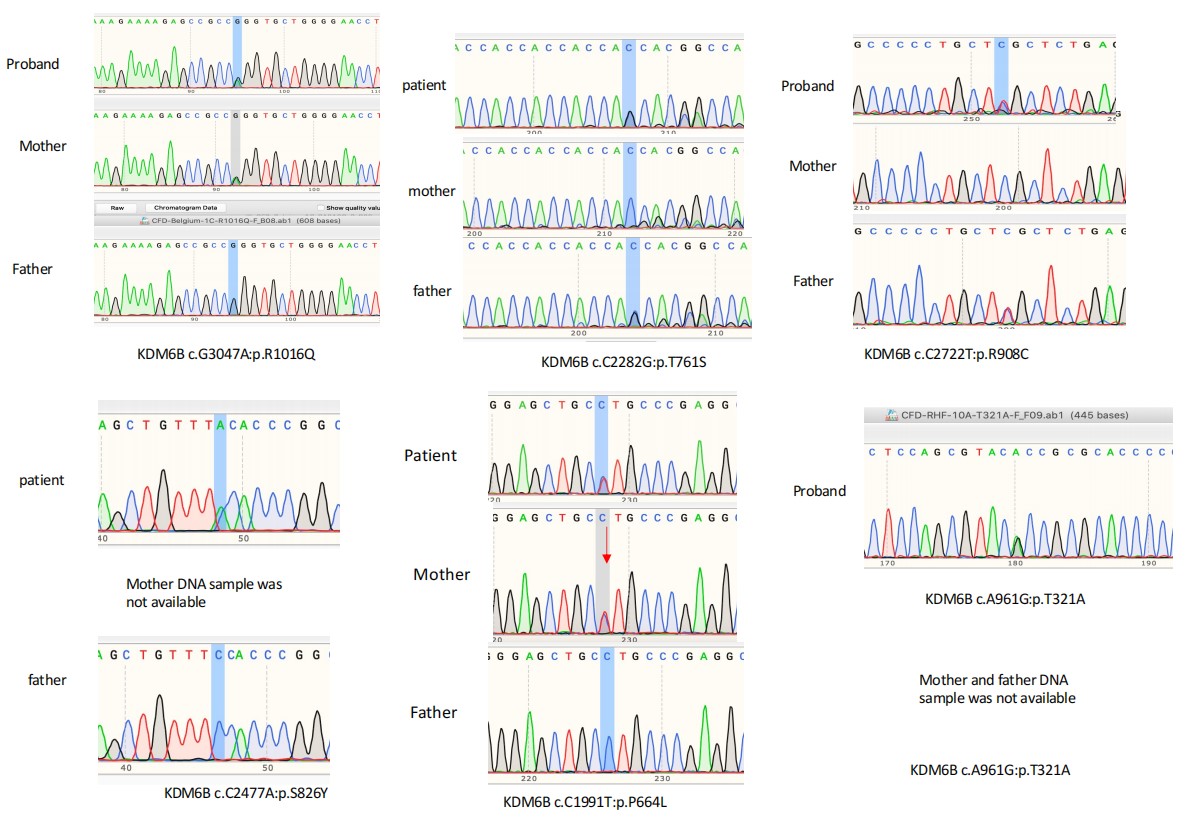

Supplement: Supplementary file 1 [file biology-12-00074-s001.zip › KDM6B Suppl Figure S1.jpg]

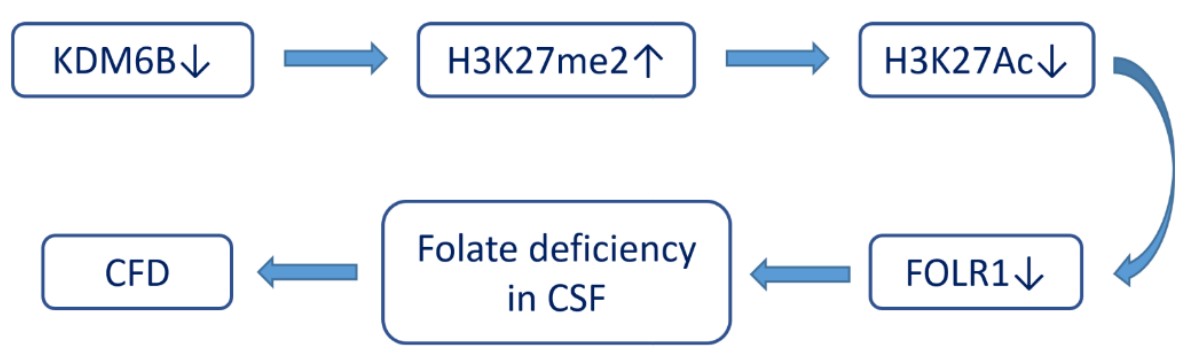

Supplement: Supplementary file 1 [file biology-12-00074-s001.zip › KDM6B Suppl Figure S2.jpg]
